# Pre-Pregnancy Body Mass Index Is Associated with Dietary Inflammatory Index and C-Reactive Protein Concentrations during Pregnancy

**DOI:** 10.3390/nu9040351

**Published:** 2017-04-01

**Authors:** Dayeon Shin, Junguk Hur, Eun-Hee Cho, Hae-Kyung Chung, Nitin Shivappa, Michael D. Wirth, James R. Hébert, Kyung Won Lee

**Affiliations:** 1 Department of Nutrition & Dietetics, College of Nursing & Professional Disciplines, University of North Dakota, Grand Forks, ND 58202, USA; dayeon.shin@und.edu; 2 Department of Biomedical Sciences, University of North Dakota School of Medicine and Health Sciences, Grand Forks, ND 58202, USA; junguk.hur@med.und.edu; 3 Division of Endocrinology and Metabolism, Department of Internal Medicine, School of Medicine, Kangwon National University, Chuncheon 24289, Korea; ehcho@kangwon.ac.kr; 4 Department of Food and Nutrition, Hoseo University, Asan 31499, Korea; hkchung@hoseo.edu; 5 Department of Epidemiology and Biostatistics, and the Cancer Prevention and Control Program, Arnold School of Public Health, University of South Carolina, Columbia, SC 29208, USA; shivappa@mailbox.sc.edu (N.S.); wirthm@mailbox.sc.edu (M.D.W.); jhebert@mailbox.sc.edu (J.R.H.); 6 Connecting Health Innovations, LLC, Columbia, SC 29201, USA; 7 Department of Food Science and Human Nutrition, Michigan State University, East Lansing, MI 48824, USA

**Keywords:** dietary inflammatory index, C-reactive protein, pregnancy body mass index, NHANES, reproductive health

## Abstract

There have been a limited number of studies examining the association between pre-pregnancy body mass index (BMI) and dietary inflammation during pregnancy. Our aim is to examine the association between pre-pregnancy BMI and the Dietary Inflammatory Index (DII)™ and C-reactive protein (CRP) concentrations during pregnancy. The study included 631 pregnant American women from the National Health and Nutrition Examination Survey (NHANES) cross-sectional examinations from 2003 to 2012. Pre-pregnancy BMI was calculated based on self-reported pre-pregnancy weight and measured height. The cut-offs of <18.5 (underweight), 18.5–24.9 (normal), 25.0–29.9 (overweight), and ≥30 kg/m^2^ (obese) were used to categorize the weight status of pregnant women prior to pregnancy. The DII, a literature-based dietary index to assess the inflammatory properties of diet, was estimated based on a one-day 24-h recall. Multivariable linear and logistic regressions were performed to estimate beta coefficients and the adjusted odds ratios (AORs) and 95% confidence intervals (95% CIs) on the association of pre-pregnancy BMI categories with the DII and CRP concentrations during pregnancy. After controlling for variables including: race/ethnicity, family poverty income ratio, education, marital status, month in pregnancy, and smoking status during pregnancy; women who were obese before pregnancy (*n* = 136) had increased odds for being in the highest tertile of the DII and CRP concentrations compared to women with normal weight (AORs 2.40, 95% CIs 1.01–5.71; AORs 24.84, 95% CIs 6.19–99.67, respectively). These findings suggest that women with pre-pregnancy obesity had greater odds of reporting higher DII and having elevated CRP. In conclusion, high pre-pregnancy BMI was associated with increased odds of pro-inflammatory diet and elevated CRP levels during pregnancy in the USA.

## 1. Introduction

Pre-pregnancy body mass index (BMI) has been associated with increased risks for adverse pregnancy outcomes such as gestational hypertension, gestational diabetes mellitus (GDM), pre-term birth, and small- and large-for-gestational-age infants [1]. Regardless of how much weight pregnant women gained during pregnancy, women with obese pre-pregnancy BMIs had increased odds for GDM compared with women with normal pre-pregnancy BMIs (adjusted odds ratios (AORs) 2.78; 95% confidence intervals (95% CIs) 2.60–2.96) [1]. Elevated inflammation during pregnancy has also been found as a risk factor for pregnancy complications such as GDM [2,3], pre-term delivery [4], and pre-eclampsia [5]. C-reactive protein (CRP), a sensitive marker of inflammation, is associated with various adverse birth outcomes during the entire pregnancy where pregnant women with elevated CRP during the first trimester had an increased risk of developing GDM [2]. In addition, elevated mid-pregnancy CRP was found in women who delivered pre-term compared to those without pre-term births [4]. 

Elevated inflammation during pregnancy may mediate the relationship between weight status before pregnancy and adverse pregnancy outcomes. It has been found that the relationship between pre-pregnancy BMI and the risk of pre-eclampsia was partially mediated by inflammation during pregnancy [6]. Elevated levels of CRP at ≤20 weeks of gestation accounted for 31% of the effect of pre-pregnancy BMI on the risk of pre-eclampsia [6]; however, the underlying mechanisms between pre-pregnancy BMI and inflammation during pregnancy are unclear. Interestingly, pre-pregnancy BMI and inflammation are both associated with dietary factors. Women with obese pre-pregnancy BMIs had significantly lower quality diets during pregnancy compared with women with normal pre-pregnancy BMIs [7,8,9]. Although there are multiple determinants for inflammation, one of the major modifiable determinants for inflammation is diet. Fruit and vegetable intake [10] and dietary fiber [11] were negatively associated with plasma CRP concentrations, which may be due to antioxidants present in fruit and vegetables [12] and decreased lipid oxidation levels [13].

The Dietary Inflammatory Index (DII)™ was developed to estimate the overall inflammatory effects of diet, and was based on an extensive literature review (around 2000 peer-reviewed articles) [14,15]. Higher DII scores were associated with increased levels of inflammation [16,17] and health outcomes such as mortality among adults in the USA [18,19], cardiovascular disease in men and women aged 55–80 years of the multicenter, randomized, nutritional intervention trial [20], lung cancer in the Melbourne Collaborative cohort study [21], laryngeal cancer [22], and lower cognitive functioning in a French population [23].

Dietary patterns considering the overall combinations of food groups and nutrients [24] among pregnant women have been studied using factor analysis or principal component analysis [25,26,27,28], reduced rank regression [29,30,31], cluster analysis [32,33], index analysis [7,8,9,34,35,36], or latent class analysis [37,38] in relation to small-for-gestational-age infants or pre-term births. To the best of our knowledge, there has been no research investigating the overall inflammatory potential of diet during pregnancy in relation to pre-pregnancy BMI. Furthermore, the association of pre-pregnancy BMI with the DII and CRP, the subjective and objective measures of inflammation, respectively, has not been explored. 

Considering that both pre-pregnancy BMI and inflammation are closely associated with dietary factors; it is important to understand how pre-pregnancy BMI is associated with diet-induced inflammation during pregnancy. If the association between pre-pregnancy BMI to inflammation is made clear, it may be possible to reduce inflammation during pregnancy through pre-pregnancy weight status. This will eventually decrease the inflammation and the risk of pregnancy complications as is the main goal under the Healthy People 2020 Maternal and Child Health initiative [39]. It is of great significance to cross-examine the relationship between pre-pregnancy BMI and inflammation during pregnancy to reduce the risk of pregnancy complications and short- and long-term adverse birth outcomes. Therefore, our aim was to examine the relationship of pre-pregnancy BMI with dietary inflammation measured by the DII and concentrations of CRP during pregnancy, and our hypothesis was that pre-pregnancy BMI is associated with the DII and CRP concentrations during pregnancy. 

## 2. Material and Methods

### 2.1. Study Population

Our study utilized public domain data from the continuous National Health and Nutrition Examination Survey (NHANES) 2003–2004, 2005–2006, 2007–2008, 2009–2010, and 2011–2012. The NHANES is a program of biennial data collection designed to assess the health and nutritional status of the civilian, non-institutionalized population of the USA, conducted by the National Center for Health Statistics (NCHS) and the Centers for Disease Control and Prevention (CDC). The NHANES uses a stratified multi-stage probability sample based on the selection of counties, blocks, households, and finally persons within households. The NHANES survey is unique in that it combines in-home interviews and physical examinations conducted at Mobile Examination Centers (MECs). Written informed consent was obtained from each survey participant for both the interview and examination. The participants were interviewed for information regarding age, race/ethnicity, education level, marital status, physical activity, and family poverty income ratio, which is a poverty measure in the poverty guidelines developed by the Department of Health Human Services (HHS) [40]. Reproductive health interviews obtained information during the gestation period at the time of the survey and pregnancy status was based on a positive urine pregnancy test. A complete description of the data-collection procedures and analytic guidelines have been provided elsewhere [41].

**Figure 1:**
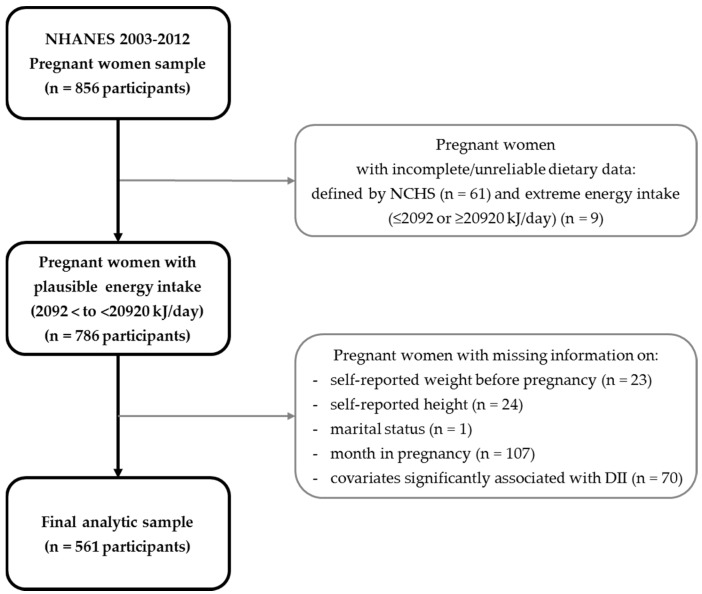
Flow chart describing National Health and Nutrition Examination Survey (NHANES) 2003–2012 pregnant women sample selection.

The 2003–2012 NHANES dataset included 856 pregnant women. Subjects were excluded if they reported incomplete or unreliable dietary data or did not meet the minimum criteria without records in individual foods files, as defined by the NCHS [42] (*n* = 61), extreme energy intake values (≤2092 kJ (500 kcal) per day and ≥20920 kJ (5000 kcal) per day, *n* = 9); or missing information on one or more of the following: self-reported weight before pregnancy (*n* = 23), self-reported height (*n* = 24), marital status (*n* = 1), or month in pregnancy (*n* = 107). The final analytic sample size was 561 pregnant women (Figure 1). The study has been reviewed and approved by the Institutional Review Board at the University of North Dakota (IRB-201610-100).

### 2.2. Exposure Variable

Pre-pregnancy BMI was calculated based on self-reported weight before pregnancy and self-reported height. Pre-pregnancy BMI from self-reported height and weight from pregnant women has been validated using the gold reference from multiple imputations [43]. The agreement in pre-pregnancy BMI classification from height and weight data between self-reported versus those measured, was high in pregnant women with the imputed weight status (*κ* = 0.78) and to the measured weight in the first trimester (*κ* = 0.76). A potential recall bias and BMI misclassification level can exist since Cohen’s kappa values were not extremely high. Self-reported pre-pregnancy BMI status was stratified into four categories based on the WHO criteria [44]: <18.5 (underweight), 18.5–24.9 (normal), 25.0–29.9 (overweight), or ≥30 kg/m^2^ (obese).

### 2.3. Outcome Variables

#### 2.3.1. Dietary Inflammatory Index (DII)

The DII was developed by researchers at the University of South Carolina. Development and validation of the DII has been published previously [15,16]. In brief, the literature (approximately 2000 articles) between 1950 and 2010 was reviewed in terms of the relationship between various micronutrients, macronutrients, and whole food items (termed food parameters) and inflammation for the purposes of deriving inflammatory effect scores of the food parameters. At the same time, DII scores were standardized to a world database, which contains the means and standard deviations of intake for these 45 food parameters from 11 populations around the world [15]. It is rare that all 45 food parameters available in any given dataset: the NHANES dietary data includes 27 DII food parameters including vitamins A, B1, B2, B3 (niacin), B6, B9 (folic acid), B12, C, D, E; iron; magnesium; zinc; selenium; carbohydrates; protein; fat; saturated, monounsaturated, and polyunsaturated fatty acids; omega-3 and omega-6 polyunsaturated fatty acids; alcohol; fiber; cholesterol; beta carotene; and caffeine. The world mean value for that food parameter was subtracted from the actual intake value for each food parameter and then divided by the world standard deviation to create a *z*-score. The next step converted the *z*-scores to percentiles using the probnorm function in SAS, which were then centered by doubling the value and subtracting one. This value was then multiplied by the inflammatory effect score for each food parameter. These were then summarized across all food parameters to derive the overall DII score. The more positive scores are more pro-inflammatory, and the negative scores are more anti-inflammatory [15]. Lastly, it should be noted that DII scores were calculated per 1000 calories consumed and a single 24-h recall was used to derive the dietary information.

#### 2.3.2. C-Reactive Protein (CRP)

CRP is an acute-phase protein produced by the liver in response to inflammation. In the NHANES 2003–2010, CRP (mg/dL) was measured by latex-enhanced nephelometry by a Behring Nephelometer for quantitative CRP determination [45,46,47,48].

### 2.4. Covariates

Multivariable models were adjusted for variables that were found to be significantly associated with the DII or pre-pregnancy BMI. Maternal age, family poverty income ratio and month in pregnancy were included in the multivariable models as continuous variables. Maternal education was grouped by the number of completed years of school: Less than high school graduate and more than college level. Race/ethnicity was divided into four groups: Hispanic, non-Hispanic white, non-Hispanic black and other (including multi-racial). Smoking status during pregnancy was defined by serum cotinine concentrations (non-smoker: ≤10 mg/L; smoker: >10 mg/L).

### 2.5. Statistical Analyses

Survey design procedures which considered the complex sampling design of NHANES were used for all analyses (SAS, version 9.4, Cary, NC, USA). Given that four cycles of 2-year data were used, 10-year sampling weights were developed by multiplying the 2-year weights by 0.2. Descriptive statistics for maternal age, family poverty income ratio, month in pregnancy, race/ethnicity, education, marital status, smoking status during pregnancy, physical activity and parity across categories of pregnancy BMI and DII tertiles were conducted with chi-square tests for categorical variables and an ANOVA test for continuous variables. Beta estimates (95% CIs) for association of pre-pregnancy BMI with the DII and CRP was calculated in both unadjusted and adjusted models. ORs and beta estimates (95% CIs) were calculated for the association between the DII and CRP in both unadjusted and adjusted models. Log-transformed CRP was used as an outcome for linear regression analyses. CRP was dichotomized at >3 mg/L and ≤3 mg/L for logistic regression analyses. 

ORs and 95% CIs were estimated using logistic regression for the association of pre-pregnancy BMI categories with the DII tertiles and the CRP tertiles, respectively, using unadjusted and adjusted models. In the multivariable model, maternal age (continuous), family poverty income ratio (continuous), month in pregnancy (continuous), race/ethnicity (Hispanic, non-Hispanic white, non-Hispanic black, and other (including multi-racial groups)), education (less than high school graduate, more than college level), and smoking status during pregnancy (yes/no) were controlled. Due to missing variables in family poverty income ratio and physical activity, there were 561 pregnant women available for multivariable analyses for the association between pre-pregnancy BMI and the DII tertiles. To investigate the association between pre-pregnancy BMI and CRP tertiles, 551 women were available with CRP values, and 528 pregnant women were available for multivariable models due to missing variables in family poverty income ratio. A test for linear trend was performed using the median approach while calculating the median for each tertile of the DII and CRP, respectively, and as a continuous variable in analyses. A two-sided *p* value < 0.05 was declared as statistically significant.

## 3. Results

The distribution of study subjects by pre-pregnancy BMI categories and tertiles of the DII is presented in Table 1. Distributions of family poverty income ratio, month in pregnancy and race/ethnicity significantly differ in the categories of pre-pregnancy BMI. Women with a level of obese pre-pregnancy BMI had low family poverty income ratios and were relatively early in their pregnancies, and were more likely be non-Hispanic black (*p* < 0.05). There were significant differences in mean DII scores in sociodemographic and lifestyle factors. Pregnant women with greater odds of having a higher DII score (i.e., more pro-inflammatory) were those with an obese pre-pregnancy BMI. Women with advanced maternal age, Hispanics and non-smokers were less likely to have a higher DII score (*p* < 0.05).

Beta estimates for the association of pre-pregnancy BMI with DII and CRP are presented in Table 2. Each kg/m^2^ increase in pre-pregnancy BMI was associated with a 0.02 (95% CIs −0.03–4.05) increase in DII during pregnancy and a 0.07 (95% CIs 0.05–0.08) increase in CRP in the covariate-adjusted model.

ORs and beta estimates for associations between the DII and CRP were calculated (Table 3), and there were no significant associations between the DII and CRP using logistic regression models. Each increase in the DII was associated with a 0.01 (95% CIs −0.04–0.06) increase in CRP in the unadjusted model and a 0.01 (95% CIs −0.03–0.06) increase in CRP in a covariate-adjusted model.

Unadjusted and adjusted ORs for the highest tertile of the DII by pre-pregnancy BMI are presented in Table 4. No significant associations were found between pre-pregnancy BMI and the DII in the unadjusted model (*p*-trend = 0.4112); however, women with obese pre-pregnancy BMIs had increased odds of being in the highest tertile of the DII (pro-inflammatory) compared to women with normal pre-pregnancy BMIs (AORs 2.40, 95% CIs 1.01–5.71, *p*-trend = 0.009), when adjusted for age, family poverty income ratio, month in pregnancy, race/ethnicity, education, and smoking status during pregnancy. Unadjusted and adjusted ORs for the highest tertile of CRP by pre-pregnancy BMIs are presented in Table 5. In the unadjusted model, women with overweight and obese pre-pregnancy BMIs had increased odds of being in the highest tertile (AORs 3.69, 95% CIs 1.21–11.24; AORs 14.67, 95% CIs 4.80–44.83, respectively, *p*-trend < 0.0001). Women with overweight and obese pre-pregnancy BMIs had increased odds for being in the highest tertile of CRP in the multivariable model (AORs 3.95, 95% CIs 1.49–10.45; AORs 24.84, 95% CIs 6.19–99.67, respectively, *p*-trend < 0.0001). 

## 4. Discussion

The present study found that pre-pregnancy BMI was associated with the DII during pregnancy in pregnant women in the USA. This was consistent with the previous finding [18] that adults who were in the highest tertile of the DII (with pro-inflammatory diets) had significantly higher BMI compared to the lowest tertile of the DII (with anti-inflammatory diets) in the NHANES III. In another study conducted by Panagos et al. [49], women with obese pre-pregnancy BMI (≥30 kg/m^2^) (*n* = 21) demonstrated a greater pro-inflammatory diet, as indicated by a higher DII score compared to women with a lean pre-pregnancy BMI (18–25 kg/m^2^) (*n* = 21) (−0.13 ± 0.82 vs. −0.68 ± 1.01, *p* = 0.06) in a follow-up study from late pregnancy (between 34 and 40 weeks gestational age) to 4–10 weeks postpartum.

As mentioned earlier, one of the most important issues relating to pre-pregnancy BMI and the DII is the quality of diet. Previous studies have examined the relationship between overall the quality of diet and pre-pregnancy BMI [7,8,9]. From the cross-sectional study of the NHANES 2003–2012, the Healthy Eating Index (HEI)-2010 was inversely associated with pre-pregnancy BMI [7]. In parallel with this finding, the cohort of Pregnancy, Infection, and Nutrition (PIN) study, the Diet Quality Index for Pregnancy (DQI-P) was inversely associated with pre-pregnancy BMI [8]. Whole grains, fruits [50], dietary fiber [51], and polyunsaturated fatty acids [52], which are major components in representative measures of the quality of diet, have shown inverse associations with inflammatory biomarkers, such as high-sensitive CRP (hs-CRP) and interleukin-6 (IL-6), in the general adult population. However, studies investigating the relationship of the quality of diet with the DII among pregnant women are limited. It would be important to cross-examine the relationship between the quality of diet and the DII during pregnancy in relation to pre-pregnancy BMI in future studies.

A strong and positive association between BMI and CRP among various populations such as non-pregnant adults and children has been well established [53,54,55]. In pregnant women, a few small-scale studies reported a positive correlation CRP with either pre-pregnancy BMI [56], or BMI at the first trimester [57]. Our present study was based on a larger study cohort (*n* = 551) and confirmed the positive significant association between pre-pregnancy BMI and CRP concentrations during pregnancy. Even after adjusting for DII, the beta estimates and standard error for the association between pre-pregnancy BMI and CRP was 0.046 and 0.007 (*p* < 0.0001), for which the underlying mechanisms are not clearly understood.

The distribution of the DII scores significantly differed according to maternal age, race/ethnicity and smoking status during pregnancy. By using multivariable logistic regressions, the DII was not significantly associated with CRP in our study, which was in contrast with previous findings where significantly increasing values of CRP were found with increasing DII scores, which in turn was associated with all-cause, cardiovascular and cancer mortality [18,58]. A pro-inflammatory diet is associated with increases in CRP concentrations and shortened telomere length in American adults [58], possibly due to elevated level of oxygen stress [59] and through the elevated level of CRP. In adults, higher DII scores significantly predicted higher plasma concentrations of IL-6 [17], tumor necrosis factor (TNF)-alpha [17], and CRP [16,17,23]. Contradicting results may be due to different target study populations such as pregnant women vs. general adults. Specifically, a lack of statistically significant difference in CRP in relation to the DII among pregnant women may be partially due to elevated inflammatory responses triggered by the progression of pregnancy [60]. 

The present study; however, has several strengths. To the best of our knowledge, this is the first study that assessed the inflammatory status based on the diets of pregnant women with respect to pre-pregnancy BMI status. Second, the present study also included CRP as well as the DII to comprehensively assess inflammatory biomarkers and the properties of diet among pregnant women. Finally, various confounding factors (including maternal sociodemographic factors and smoking status during pregnancy) were controlled when examining the relationship of pre-pregnancy BMI with the DII during pregnancy.

There were also a few notable limitations in this study. First, only one 24-h recall was used to assess dietary information in the analysis. Using only one day of dietary information may not have accounted for the day-to-day variability in diet, leading to imprecise estimates [61]; Second, the relatively small sample size, particularly the number of underweight pregnant women, may be one of the limitations of this cross-sectional study. It may have been difficult to detect the significant difference on the DII and inflammatory biomarker, CRP across all the pre-pregnancy BMI categories. Third, potential under-reporting of dietary intakes by obese women might have occurred. Additionally, diets and metabolic profiles may have evolved during pregnancy. Fourth, although sociodemographic factors and pre-pregnancy BMI did not significantly differ between two groups of pregnant women with or without CRP values (631 vs. 551), the DII was significantly lower in women without a CRP value compared to those with CRP values (−0.2 ± 0.3 vs. 0.7 ± 0.2). Finally, due to the cross-sectional study design, the cause-effect relationship between BMI before pregnancy and the DII during pregnancy could not be drawn from this study.

## 6. Conclusions

In conclusion, high pre-pregnancy BMI was associated with increased risks of pro-inflammatory diet and elevated CRP levels in pregnant women. Future research is warranted to explore whether increased inflammation in obese pre-pregnancy women may adversely affect offspring health in the long-term. 

## Figures and Tables

**Table 1:** Distributions of socio-demographics and lifestyle factors by pre-pregnancy body mass index (BMI) and Dietary Inflammatory Index (DII) categories.

Socio-Demographics and Lifestyle Factors	Pre-Pregnancy BMI ^1^									DII						
	Underweight		Normal		Overweight		Obese		*p* Value	Tertile 1		Tertile 2		Tertile 3		*p* Value
	(*n* = 31)		(*n* = 311)		(*n* = 153)		(*n* = 136)			(*n* = 210)		(*n* = 211)		(*n* = 210)		
	Mean	SEM	Mean	SEM	Mean	SEM	Mean	SEM		Mean	SEM	Mean	SEM	Mean	SEM	
Maternal Age (years)	27.5	1.4	28.1	0.6	28.5	0.9	28.1	0.8	0.8520	30.2	0.8	27.4	0.6	26.5	0.6	<0.0001
Family Poverty Income Ratio (*n* = 601)	2.5	0.5	3.1	0.2	2.7	0.3	2.3	0.2	0.0013	3.1	0.2	2.7	0.2	2.6	0.2	0.0775
Month in Pregnancy	5.2	0.7	5.9	0.3	5.6	0.3	4.7	0.3	0.0037	5.8	0.3	5.2	0.2	5.5	0.3	0.3511
	*n*	(Wt’d % ^2^)	*n*	(Wt’d %)	*n*	(Wt’d %)	*n*	(Wt’d %)		*n*	(Wt’d %)	*n*	(Wt’d %)	*n*	(Wt’d %)	
Race/Ethnicity																
Hispanic	4	2.0	93	47.4	52	26.7	48	23.9	<0.0001	80	43.4	75	35.0	42	21.6	0.0002
Non-Hispanic white	17	3.3	152	55.8	67	18.9	48	22.0		91	36.3	85	28.3	108	35.4	
Non-Hispanic black	4	5.0	39	25.8	25	19.4	37	49.8		17	15.4	39	38.9	49	45.7	
Other (including multi-racial)	6	16.6	27	63.2	9	15.8	3	4.4		22	56.7	12	31.4	11	11.9	
Education																
≤High school graduate	12	4.4	140	42.8	85	24.5	77	28.4	0.2622	91	29.5	108	34.0	115	36.5	0.2086
≥College	19	4.5	171	54.6	68	17.9	59	23.0		119	40.4	103	30.2	95	29.4	
Marital Status																
Married/living with partner	26	4.6	244	51.4	122	21.2	94	22.8	0.3537	181	38.6	168	32.0	137	29.4	0.2410
Widowed/divorced/separated/single	5	3.8	67	45.8	31	16.8	42	33.5		29	27.7	43	29.8	73	42.5	
Smoking Status during Pregnancy ^3^ (*n* = 585)																
No	26	4.0	255	53.5	127	18.2	109	24.3	0.4225	185	38.7	173	29.5	159	31.8	0.0016
Yes	3	8.5	38	42.9	12	28.0	15	20.5		8	10.1	22	35.0	38	54.9	
Physical Activity (*n* = 354)																
Light (0–500 MET ^4^-min/week)	10	3.8	88	53.8	48	20.4	41	22.0	0.8083	72	39.6	47	24.3	68	36.1	0.9646
Moderate (500–1000 MET-min/week)	3	2.9	49	54.2	12	28.6	12	14.3		30	44.2	28	26.5	18	29.3	
Active (≥1000 MET-min/week)	3	2.4	53	64.0	21	18.2	14	15.4		36	45.7	30	20.5	25	33.8	
Parity (*n* = 297)																
None	0	.	11	77.2	2	8.1	3	14.7	n/a	3	11.6	5	39.5	8	48.9	0.2143
1	7	3.0	73	49.1	37	23.1	31	24.8		53	28.7	55	41.0	40	30.4	
2	3	4.3	35	56.1	25	15.7	21	23.9		33	49.1	26	24.1	25	26.8	
≥3	0	.	26	61.5	15	29.2	8	9.3		16	43.0	14	22.3	19	34.7	

^1^ Pre-pregnancy BMI was stratified into four categories based on the WHO criteria: <18.5 kg/m^2^ (underweight), 18.5–24.9 kg/m^2^ (normal), 25.0–29.9 kg/m^2^ (overweight), and ≥30 kg/m^2^ (obese); ^2^ Wt’d % = Weighted percentage. Sample weights were created in NHANES to account for the complex survey design (including oversampling of some subgroups), survey non-responses, and post-stratification. When a sample was weighted in NHANES, it was representative of the US civilian non-institutionalized census population; ^3^ Smoking status during pregnancy was defined by serum cotinine concentrations (non-smoker: ≤10 mg/L; smoker >10 mg/L); ^4^ MET (Metabolic Equivalent of Task): Total MET-min/week from self-reported leisure-time physical activities. Tertile 1 was the highest anti-inflammatory group, and Tertile 3 was the most pro-inflammatory group. DII ranges for Tertile 1, Tertile 2 and Tertile 3 were −4.98–0.07, 0.08–1.67 and 1.68–4.14, respectively. *p* value: ANOVA test for continuous variables, and Chi-square test for categorical variables. n/a: Not available.

**Table 2:** Beta estimates for association of pre-pregnancy BMI with DII and C-reactive protein (CRP).

	DII (*n* = 631 ^1^; 561 ^2^)	CRP ^3^ (*n* = 551 ^1^; 528 ^2^)
	Beta (95% CIs)	Beta (95% CIs)
Pre-pregnancy BMI ^4^		
Unadjusted	0.03 (−0.35–0.90)	0.06 (0.05–0.07)
Multivariable ^5^	0.02 (−0.03–4.05)	0.07 (0.05–0.08)

^1^ Unadjusted model; ^2^ Multivariable model; ^3^ CRP was log-transformed; ^4^ Pre-pregnancy BMI (kg/m^2^) was calculated as a continuous variable; ^5^ Adjusted for age (continuous), family poverty income ratio (continuous), month in pregnancy (continuous), race/ethnicity (Hispanic, non-Hispanic white, non-Hispanic black, other (including multi-racial)), education (≤high school graduate, ≥college) and smoking status during pregnancy (yes/no).

**Table 3:** Odds ratio and beta estimates for associations between DII and CRP.

	CRP (*n* = 551 ^1^; 528 ^2^)	
	OR (95% CIs) ^3^	Beta (95% CIs) ^4^
DII continuous		
Unadjusted	0.97 (0.77–1.22)	0.01 (−0.04–0.06)
Multivariable ^5^	0.94 (0.75–1.19)	0.01 (−0.03–0.06)

^1^ Unadjusted model; ^2^ Multivariable model; ^3^ CRP was dichotomized at >0.3 mg/dL vs. ≤0.3 mg/dL for logistic regression analyses; ^4^ CRP was log-transformed; ^5^ Adjusted for age (continuous), family poverty income ratio (continuous), month in pregnancy (continuous), race/ethnicity (Hispanic, non-Hispanic white, non-Hispanic black, other (including multi-racial)), education (≤high school graduate, ≥college) and smoking status during pregnancy (yes/no).

**Table 4:** Unadjusted and adjusted odds ratios (ORs) and 95% CIs g in the highest DII tertile by pre-pregnancy BMI categories.

	Unadjusted (*n* = 631)				^1^ Adjusted (*n* = 561)		
	Tertile 3 vs. Tertile 1 (Reference)				Tertile 3 vs. Tertile 1 (Reference)		
	ORs	95% CIs			AORs	95% CIs	
Pre-pregnancy BMI				Pre-pregnancy BMI			
Underweight	2.26	0.58	8.85	Underweight	3.11	0.85	11.45
Normal	1.00			Normal	1.00		
Overweight	1.31	0.56	3.11	Overweight	1.44	0.56	3.73
Obese	2.15	0.96	4.83	Obese	2.40	1.01	5.71
*p* trend ^2^	0.4112			*p* trend ^2^	0.009		
*p* trend ^3^	0.116			*p* trend ^3^	0.037		
				Age (continuous)	0.89	0.83	0.96
				Family Poverty Income Ratio (continuous) (*n* = 601)	1.11	0.81	1.53
				Month in Pregnancy (continuous)	0.98	0.81	1.18
				Race/Ethnicity			
				Hispanic	0.45	0.15	1.32
				Non-Hispanic white	1.00		
				Non-Hispanic black	2.30	0.63	8.45
				Other (including multi-racial groups)	0.37	0.11	1.18
				Education			
				≤High school graduate	1.68	0.60	4.71
				≥College	1.00		
				Smoking Status during Pregnancy ^4^ (*n* = 585)			
				Yes	4.25	1.25	14.51
				No	1.00		

Mean ± SE for Tertile 1 (reference), Tertile 2 and Tertile 3 was –1.6 ± 0.1, 1.0 ± 0.1 and 2.4 ± 0.1, respectively. ^1^ Adjusted for age (continuous), family poverty income ratio (continuous), month in pregnancy (continuous), race/ethnicity (Hispanic, non-Hispanic white (reference), non-Hispanic black, other including multi-racial), education (≤high school graduate, ≥college (reference)) and smoking status during pregnancy (yes/no). *p* trend was obtained by ^2^ using the median approach, calculating median for each tertile of the DII as a continuous variable in analyses and ^3^ treating each DII as a continuous variable in the linear regression model. ^4^ Smoking status during pregnancy was defined by serum cotinine concentrations (non-smoker: ≤10 mg/L (reference); smoker >10 mg/L). Self-reported pre-pregnancy BMI was stratified into four categories based on the WHO criteria: <18.5 (underweight, reference), 18.5–24.9 (normal), 25.0–29.9 (overweight), and ≥30 kg/m^2^ (obese).

**Table 5:** Unadjusted and adjusted odds ratios (ORs) and 95% CIs for being in the highest CRP tertile by pre-pregnancy BMI categories.

	Unadjusted (*n* = 551)				Adjusted ^1^ (*n* = 528)		
	Tertile 3 vs. Tertile 1 (Reference)				Tertile 3 vs. Tertile 1 (Reference)		
	ORs	95% CIs			AORs	95% CIs	
Pre-pregnancy BMI				Pre-pregnancy BMI			
Underweight	0.25	0.06	1.08	Underweight	0.36	0.08	1.56
Normal	1.00			Normal	1.00		
Overweight	3.69	1.21	11.24	Overweight	3.95	1.49	10.45
Obese	14.67	4.80	44.83	Obese	24.84	6.19	99.67
*p*-trend ^2^	<0.0001			*p*-trend ^2^	<0.0001		
*p*-trend ^3^	<0.0001			*p*-trend ^3^	<0.0001		
				Age (continuous)	1.02	0.95	1.10
				Family Poverty Income Ratio (continuous) (*n* = 528)	1.41	1.07	1.85
				Month in Pregnancy (continuous)	1.13	0.98	1.30
				Race/Ethnicity			
				Hispanic	2.62	1.14	6.03
				Non-Hispanic white	1.00		
				Non-Hispanic black	2.04	0.76	5.47
				Other (including multi-racial groups)	1.88	0.22	16.12
				Education			
				≤High school graduate	3.45	1.17	10.20
				≥College	1.00		
				Smoking Status during Pregnancy ^4^			
				Yes	1.91	0.61	6.00
				No	1.00		

^1^ Adjusted for age (continuous), family poverty income ratio (continuous), month in pregnancy (continuous), race/ethnicity (Hispanic, non-Hispanic white (reference), non-Hispanic black, other (including multi-racial)), education (≤high school graduate, ≥college (reference)) and smoking status during pregnancy (yes/no). *p*-trend was obtained by ^2^ using the median approach, calculating median for each tertile of CRP values as a continuous variable in analyses and ^3^ treating each DII as a continuous variable in the linear regression model. ^4^ Smoking status during pregnancy was defined by serum cotinine concentrations (non-smoker: ≤10 mg/L (reference); smoker >10 mg/L). Self-reported pre-pregnancy BMI was stratified into four categories based on the WHO criteria: <18.5 (underweight), 18.5–24.9 (normal, reference), 25.0–29.9 (overweight), and ≥30 kg/m^2^ (obese).
